# Impact of body mass index and fat distribution on sex steroid levels in endometrial carcinoma: a retrospective study

**DOI:** 10.1186/s12885-019-5770-6

**Published:** 2019-06-07

**Authors:** Willem Jan van Weelden, Kristine Eldevik Fasmer, Ingvild L. Tangen, Joanna IntHout, Karin Abbink, Antionius E. van Herwaarden, Camilla Krakstad, Leon F. A. G. Massuger, Ingfrid S. Haldorsen, Johanna M. A. Pijnenborg

**Affiliations:** 1 Department of Obstetrics and Gynecology, Radboud University Medical Center, Geert Grooteplein 10, P.O. Box 9101, 6500 HB Nijmegen, The Netherlands; 2 Mohn Medical Imaging and Visualization Centre, Department of Radiology, Haukeland University Hospital Bergen, Bergen, Norway; 3 Department of Radiology, Department of Clinical Medicine, University of Bergen, Bergen, Norway; 4 Department of Gynecology and Obstetrics, Haukeland University Hospital, Bergen, Norway; 5 Centre for Cancer Biomarkers, Department of Clinical Science, University of Bergen, Bergen, Norway; 6 Department for Health Evidence, Radboud University Medical Center, Nijmegen, Netherlands; 7 Department of Laboratory Medicine, Radboud University Medical Center, Nijmegen, Netherlands

**Keywords:** Endometrial cancer, Obesity, Estradiol, Subcutaneous fat, Visceral fat

## Abstract

**Background:**

Obesity is an important cause of multiple cancer types, amongst which endometrial cancer (EC). The relation between obesity and cancer is complicated and involves alterations in insulin metabolism, response to inflammation and alterations in estradiol metabolism. Visceral obesity is assumed to play the most important role in the first two mechanisms, but its role in estradiol metabolism is unclear. Therefore, this retrospective study explores the relationship of body mass index (BMI), visceral fat volume (VAV) and subcutaneous fat volume (SAV) and serum levels of sex steroids and lipids in patients with endometrial cancer.

**Methods:**

Thirty-nine postmenopausal EC patients with available BMI, blood serum and Computed Tomography (CT) scans were included. Serum was analyzed for estradiol, dehydroepiandrosterone sulfate (DHEAS), androstenedione, testosterone, cholesterol, triglycerides and high (HDL), low (LDL) and non-high density (NHDL) lipoprotein. VAV and SAV were quantified on abdominal CT scan images. Findings were interpreted using pearson correlation coefficient and linear regression with commonality analysis.

**Results:**

Serum estradiol is moderately correlated with BMI (r = 0.62) and VAV (r = 0.58) and strongly correlated with SAV (r = 0.74) (*p* < 0.001 for all). SAV contributes more to estradiol levels than VAV (10.3% for SAV, 1.4% for VAV, 35.9% for SAV and VAV, *p* = 0.01). Other sex steroids and lipids have weak and moderate correlations with VAV or SAV.

**Conclusions:**

This study shows that serum estradiol is correlated with BMI and other fat-distribution measures in postmenopausal endometrial cancer patients. Subcutaneous fat tissue contributes more to the estradiol levels indicating that subcutaneous fat might be relevant in endometrial cancer carcinogenesis.

**Electronic supplementary material:**

The online version of this article (10.1186/s12885-019-5770-6) contains supplementary material, which is available to authorized users.

## Background

Endometrial cancer (EC) is the most common gynecological cancer in developed countries with an annual incidence rate of 320,000 cases world-wide [1]. Obesity is the most important risk factor for EC as current estimates attribute 40% of new EC cases to obesity [2, 3]. Consequently, the increase in obesity has caused a concurrent increase in EC incidence in the last decades [4–6]. The biological relation between obesity and cancer is complex, and involves alterations in insulin metabolism, inflammatory response and sex steroid metabolism [7, 8].

EC occurs predominantly in postmenopausal women in which estrogen formation mainly depends on the aromatase activity in fat [9, 10]. Androstenedione and testosterone are the main substrates for aromatization into estrone and estradiol (E2). Dehydroepiandrosterone sulfate (DHEAS) produced by the adrenal gland is the main source of androstenedione and testosterone. Although E2 is more potent than E1, E1 is the most abundant form of estrogen in postmenopausal women. E2 is either synthesized directly from testosterone by aromatase, or through reduction of E1 [9]. Estradiol induces endometrial proliferation which, in the absence of progesterone, may lead to endometrial cancer [7, 8]. EC is traditionally classified into endometrioid (EEC) and non-endometrioid endometrial cancer (NEEC). EEC carcinogenesis is driven by estradiol, while NEEC is assumed to develop independent of estrogens, although recent data suggests that estradiol might also play a role in NEEC tumorigenesis [11–13].

Obesity is quantified with the body mass index (BMI), yet BMI does not reflect the fat distribution in the visceral abdominal volume (VAV) and subcutaneous abdominal volume (SAV). VAV is assumed to be metabolically more active than SAV, and is involved in production of cytokines and release of free fatty acids that contribute to inflammation and alterations in insulin metabolism associated with metabolic syndrome and risk of EC [8, 14]. However, the role of VAV and SAV in the production of sex steroids and lipids in EC patients is unknown [15–17]. VAV and SAV can reliably be quantified using abdominal computed tomography (CT) scans [18]. Previous studies have reported a relation between SAV and development of EC, while VAV was associated with adverse outcome [19–21]. However, neither serum sex steroids nor lipid levels were measured in these studies. Therefore the aim of this study was to explore the relation between BMI, visceral and subcutaneous fat volumes and serum levels of sex steroids and lipids in endometrial cancer patients.

## Methods

### Aim, design and setting

The objective of this study was to explore the relation between BMI, visceral and subcutaneous fat volumes and serum levels of sex steroids and lipids in endometrial cancer patients. To that end, a retrospective study was performed in the Radboud university medical center, a tertiary gynecologic oncology center in the Netherlands.

### Patients

All surgically treated postmenopausal patients with endometrial cancer were eligible if they had no history of hormonal treatment or contraceptive use within 3 months prior to diagnosis. From the Radboudumc biobank, a total of 48 postmenopausal EC patients treated between 1999 and 2009 was included. Nine patients were excluded: three because no preoperative serum was available and six because no preoperative CT was available, leaving 39 patients for analysis. All patients underwent primary hysterectomy with bilateral salpingo-oophorectomy and lymphadenectomy was performed if indicated [22]. Patients were staged according to the International Federation of Gynaecology and Obstetrics (FIGO) staging criteria [23]. Adjuvant radiotherapy or chemotherapy was given in case of high risk factors. Tumours were classified into EEC and NEEC and reviewed by an expert pathologist (J.B.).

Baseline characteristics were extracted from patient records including patient and tumor characteristics and CT-scan results. FIGO stage I-II was defined as early stage and FIGO stage III-IV as advanced stage. BMI was based on measured weight and height or patient reported weight and height at diagnosis. The study was carried out in accordance with the Declaration of Helsinki and was approved by the Local Ethical Committee of Radboudumc. All patients gave written informed consent for use of clinical data and tissue before entry in the Radboudumc biobank.

### Fat distribution

**Fig. 1:**
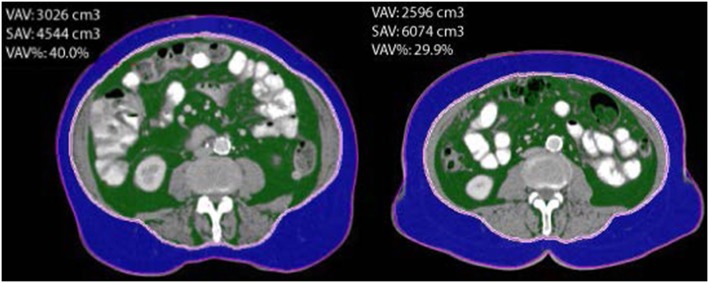
Representative examples of CT scan results. Visceral fat in green and subcutaneous fat in purple. CT scan on the left with high VAV, CT scan on the right with high SAV

CT scan images were analyzed at the University Hospital Bergen, Norway using a semi-automated method for volumetric quantification of abdominal fat (iNtuition software program, TeraRecon Inc., San Mateo, CA, USA). This program estimates VAV and SAV based on cross-sectional CT scan images from the upper right diaphragm to L5/S1 level using segmentation of pixels with values for Hounsfield units (HU) corresponding to fat tissue (− 195 to − 45 HU) [18]. The correct segmentation of SAV and VAV was visually verified by the operator (W.W.), and manually adjusted if necessary as illustrated in Fig. 1. The sum of SAV and VAV was considered to comprise the total abdominal fat volume (TAV, cm^3^). The percentage of visceral out of total abdominal fat volume ([VAV/TAV]× 100; VAV%) was also calculated. Waist circumference (WC) was measured at the level of vertebral body L3/L4.

### Serum sex steroid and lipid levels

Non fasting blood samples were collected according the Radboudumc biobank protocol, at the last outpatient visit prior to surgery. Blood samples were collected in vacutainer tubes, centrifuged at 200 *g* during 10 min and stored at − 40 °C until assayed. Analyses of sex steroids included DHEAS, testosterone, androstenedione (A4) and E2. The following lipids were analyzed: cholesterol, high (HDL), low, (LDL) and non-high density lipoprotein (NHDL) and triglycerides. DHEAS and E2 (3th generation) were measured by ECLIA (Electro-chemiluminescence immunoassay) on a Modular E170 random access analyzer (Roche, Basel, Switzerland). A4 and T were measured using an in-house developed liquid-chromatography tandem mass spectrometry (LC-MS/MS) [24]. In case sample measurements were below the detection level, calculations were performed using the lower limit of detection divided by 2. Cholesterol (2nd generation), triglycerides and HDL (3rd generation) were analyzed by an enzymatic colorimetric method on a Cobas 6000 random access analyser (Roche, Basel, Switzerland). LDL was calculated with the Friedewald formula. NHDL was calculated by subtracting HDL from cholesterol. All samples were analyzed on the same day.

### Statistical analysis

Differences in baseline characteristics, fat distribution values and serum levels between EEC and NEEC were compared using the χ^2^ test for discrete variables and the Mann Whitney U test for continuous variables. The correlations between log transformed sex steroid and lipid levels and fat measures were analyzed using Spearman rho’s for not-normally distributed variables and visualized with scatterplots and regression lines. Correlations of r > 0.7 were considered strong, correlations between r = 0.4 and r = 0.7 as moderate and correlations of r < 0.4 as weak [25, 26]. Linear regression was followed by commonality analysis in order to identify the unique and common contribution of the independent variables to the variation of the dependant variable [27]. All tests were two-sided and *p*-values of < 0.05 were considered significant. Statistical analyses were performed using SPSS (version 22.0 for Microsoft, SPSS Inc., Chicago, IL.) and R (version 3.5.1 https://www.r-project.org), using the yhat package version 2.0.0 [28, 29]. As this study does not report on clinical outcome, no relevant core outcome set was available for this research (http://www.crown-initiative.org/core-outcome-sets/).

## Results

### Patients

Clinicopathological characteristics of the study cohort (*n* = 39) are summarized in Table 1. The median age at diagnosis was 68 years and the median BMI was 26.9 kg/m^2^. Patients with NEEC histology (*n* = 14) were more frequently diagnosed with advanced stage (71% versus 28%) and recurrence (58% versus 24%) compared to patients with EEC histology. There was no significant difference in EC related mortality between patients with EEC and NEEC.

**Table 1:** Clinicopathological characteristics of study cohort

	Total, *n* = 39	EEC, *n* = 25	NEEC, *n* = 14	p
Age (years)^a^	68 (50–88)	68 (50–85)	74,5 (54–82)	0.098
BMI (kg/m^2^)^a^	26.9 (20–51)	27.5 (20–51)	25.7 (22–37)	0.289
Follow-up (months)^a^	34 (1–168)	34 (1–88)	30 (2–168)	0.946
Hypertension^b^				0.133
Yes	17	9 (36%)	8 (62%)	
No	21	16 (64%)	5 (38%)	
Diabetes mellitus^b^				0.825
Yes	8 (21%)	5 (20%)	3 (23%)	
No	30 (79%)	20 (80%)	10 (77%)	
Tumor grade				
1	4 (10%)	4 (16%)		
2	16 (41%)	16 (64%)		
3	19 (48%)	5 (20%)	14 (100%)	
Histology				
Endometrioid	25 (64%)	25 (64%)		
Non-endometrioid	14 (36%)		14 (36%)	
Serous	2 (5%)		2 (5%)	
Clear cell	2 (5%)		2 (5%)	
Mixed	5 (13%)		5 (13%)	
Carcinosarcoma	5 (13%)		5 (13%)	
FIGO stage				0.009^*^
Early (I-II)	22 (56%)	18 (72%)	4 (29%)	
Advanced (III-IV)	17 (44%)	7 (28%)	10 (71%)	
Recurrence^c^				0.041^*^
Yes	13 (33%)	6 (24%)	7 (58%)	
No	24 (62%)	19 (76%)	5 (42%)	
EC related death				0.542
Yes	9 (23%)	5 (20%)	4 (29%)	
No	30 (77%)	20 (80%)	10 (71%)	

*significant result, ^a^ Median value (range) ^b^ data missing in one patient, ^c^ data missing in two patients

### Correlation of sex steroid- and lipid levels with abdominal fat volumes

The correlation coefficients of estradiol in relation to fat volumes showed strong correlations with SAV (r = 0.74, *p* < 0.01) and TAV (r = 0.74, *p* < 0.01) and moderate correlations with BMI (r = 0.62, *p* < 0.01), WC (r = 0.64, *p* < 0.01) and VAV (r = 0.58, p < 0.01) (Table 2). A4 had a moderate correlation with SAV (r = 0.43, *p* < 0.01) and had weak correlations with WC and TAV (r = 0.33 and 0.37, both *p* < 0.05). DHEAS was weakly correlated with BMI, VAV and SAV (r = 0.36, r = 0.35 and 0.34, all *p* < 0.05; Table 2).

**Table 2:** Correlation coefficients of steroid and lipid serum levels and fat measurements

	BMI	WC	TAV	VAV	SAV^a^	VAV%^b^
Sex steroids						
Estradiol (pmol/L)	.62**	.64**	.74**	.58**	.74**	−.06
Androstenedione (nmol/L)	.26	.33*	.37*	.29	.43**	−.17
Testosterone (nmol/L)	.17	.31	.29	.19	.31	−.15
DHEAS (μmol/L)	.36*	.31	.30	.35*	.34*	−.10
Lipids						
Cholesterol (mmol/L)	−.07	−.08	−.19	−.08	−.20	.01
HDL (mmol/L)	−.23	−.37*	−.45**	−.39*	−.42**	−.15
LDL (mmol/L)	−.03	−.03	−.13	−.02	−.12	−.01
NHDL (mmol/L)	−.00	.01	−.08	.01	−.09	.05
Triglycerides (mmol/L)	−.05	.17	.15	.14	.09	.36*
Chol/HDL ratio	.17	.27	.29	.30	.27	--.13

^a^ SAV analysis is limited to 38 patients, ^b^ proportion of VAV in relation to TAV, * *p* < 0.05, **p < 0.01

HDL had a moderate negative correlation with TAV (r = − 0.45, *p* < 0.01) and SAV (r = − 0.42, *p* < 0.01) and a weak negative correlation with WC (r = − 0.37, *p* < 0.05) and VAV (r = − 0.39, *p* < 0.05). Triglycerides showed a weak correlation with the proportion of VAV in relation to TAV (VAV%) (r = 0.36, *p* < 0.05), but not with VAV or SAV (Table 2).

Within the individual sex steroids there were moderate correlations between A4 and DHEAS and A4 and testosterone (Additional file 1: Table S1). Strong correlations between lipids occurred between cholesterol and LDL, cholesterol and NHDL, and LDL and NHDL (Additional file 1: Table S2). Correlations of fat distribution measures are shown in Additional file 1: Table S3.

### Sex steroids, lipids and fat distribution in endometrioid and non-endometrioid histology

No significant differences in fat distribution measurements were observed between patients with EEC and NEEC (Table 3). The EEC subgroup had significantly higher serum levels of cholesterol, LDL and NHDL compared to the NEEC group without significant differences in sex steroid levels (Table 3).

**Table 3:** Fat distribution measurements and serum levels of study cohort

	Total, *n* = 39 Median (SD)	EEC, *n* = 25 Median (SD)	NEEC, *n* = 14 Median (SD)
Fat distribution			
BMI (kg/m^2^)	26.9 (7.9)	27.5 (9.0)	26.1 (4.7)
WC (cm)	95.3 (15.8)	97.8 (17.9)	93 (11.0)
TAV (cm^3^)	7529 (4633)	7487 (5161)	7570 (3484)
VAV (cm^3^)	2772 (1677)	2861 (1741)	2668 (1610)
SAV (cm^3^)	4544 (3230)	4556 (3669)	4544 (2181)
VAV% (%)	33.4 (8.2)	32.5 (8.3)	34.4 (8.2)
Sex steroids			
Estradiol (pmol/L)	54 (38.4)	49 (43.8)	56 (27.2)
Androstenedione (nmol/L)	1.8 (1.1)	1.8 (1.1)	1.7 (1.2)
Testosterone (nmol/L)	0.7 (0.6)	0.7 (0.6)	0.6 (0.4)
DHEAS (μmol/L)	2.4 (1.5)	2.4 (1.3)	2.31 (1.8)
Lipids			
Cholesterol (mmol/L)	5.1 (1.4)	5.8 (1.3)*	3.8 (1.4)*
HDL (mmol/L)	0.8 (0.4)	0.8 (0.4)	0.6 (0.4)
LDL (mmol/L)	3.5 (1.2)	3.8 (1.1)*	2.6 (1.2)*
NHDL (mmol/L)	4.3 (1.3)	4.5 (1.3)*	3.2 (1.3)*
Triglycerides (mmol/L)	1.5 (0.6)	1.5 (0.6)	1.5 (0.4)
Chol/HDL ratio	7 (3.4)	7 (3.3)	8 (3.5)

*significant differences at *p* < 0.05

**Fig. 2:**
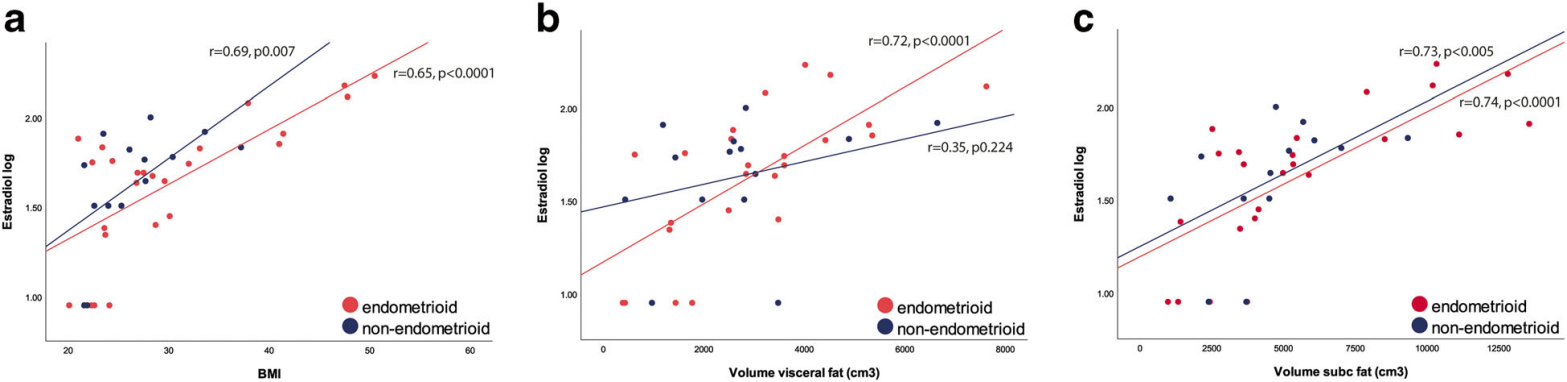
Correlation of estradiol and fat distribution measures in EEC and NEEC. **a**: correlation of estradiol and BMI, **b**: correlation of estradiol and VAV, **c**: correlation of estradiol and SAV

Estradiol had a moderate correlation with BMI in both EEC and NEEC patients (r = 0.65, p0.007 and 0.69, *p* < 0.0001; Fig. 2). Estradiol was strongly correlated with VAV (r = 0.72, *p* < 0.0001) in EEC, but had only a weak correlation in NEEC (r = 0.35, p0.224; Fig. 2), whereas SAV was strongly correlated in both EEC and NEEC patients (r = 0.74, *p* < 0.005 and 0.73, *p* < 0.0001; Fig. 2).

### Contribution of VAV and SAV to production of estradiol

Based on the commonality analysis in the multiple linear regression model of estradiol in relation to SAV and VAV, most of the variation in estradiol levels was explained by SAV and VAV together, due to the strong correlation between SAV and VAV (r = 0.85, *p* < 0.001). In the study cohort, the total explained variation in estradiol levels was 47.6%: the unique contribution of SAV was 10.3% (significant at *p* = 0.01), VAV explained 1.4% (*p* = 0.33) [27], and the common contribution was 35.9%. There was no significant difference between the EEC and NEEC subgroups (*p* = 0.64).

## Discussion

The link between obesity and endometrial cancer is explained by three potential mechanisms: (1) secretion of free fatty acids leading to increased levels of insulin and insulin growth factor, (2) production of cytokines that lead to a persistent inflammatory environment and (3) alterations in estradiol metabolism. In the current study a positive correlation was observed between serum estradiol levels and BMI and other fat measures in endometrial cancer patients. Furthermore, we observed a stronger correlation of subcutaneous fat volume (SAV) with estradiol when compared to visceral fat volume (VAV). Finally, SAV was more contributive to the estradiol levels than VAV.

Estradiol plays an important role in the oncogenesis of hormone dependent cancers like postmenopausal breast cancer and endometrial cancer through its proliferative activity in the target tissue. In endometrial cancer, the absence of counteractive effects of progesterone on the endometrium in postmenopausal women, leads to endometrial hyperplasia and eventually endometrioid type endometrial cancer [11]. The role of estradiol is supported by the higher incidence of EC observed in patients with exogenous estrogen use, and is in line with the known endogenous estrogen related risk factors i.e. nulliparity, early menarche, late menopause and high BMI [30–32]. Recently, EC patients were shown to have higher serum levels of estradiol compared to healthy controls [17, 33]. Our study is the first to evaluate the correlation of fat measures with serum estradiol in EC patients. Additionally, our results indicate that SAV has a more important role in estradiol production than VAV. *Hetemaki* et al. and *Wang* et al. have reported that estradiol levels within VAV and SAV did not differ in studies in healthy postmenopausal and obese premenopausal women [15]. However, *Wang* et al also showed a higher *CYP19A1* expression in SAV compared to VAV among obese premenopausal women undergoing bariatric surgery. *CYP19A1* encodes for the aromatase enzyme, which suggest that a higher aromatase activity could explain the more important role of SAV in estradiol metabolism [34].

Several studies have suggested a synergistic activity of estradiol and insulin in the endometrioid endometrial carcinogenesis [8, 35, 36]. Alterations in insulin metabolism are mainly mediated by VAV [37, 38]. Combining this with our results suggests that both SAV and VAV are relevant for EC carcinogenesis: SAV is primarily responsible for estradiol production and VAV is involved in alterations leading to hyperinsulinemia.

With respect to NEEC carcinogenesis, it is interesting to observe the similar relation between BMI and estradiol in EEC and NEEC patients. Up till now, the role of estradiol in NEEC carcinogenesis is largely unknown [13, 39–41]. Estradiol might contribute to NEEC development directly through stimulation of estradiol responsive parts of heterogenic non-endometrioid tumors, or indirectly by obesity associated mechanisms such as alterations in the insulin metabolism or in the inflammatory response [7, 8].

Strengths of this study include the selection of EC patients with different tumor grades, stages and histology. Due to the standardized assessment of fat compartments on CT scans, a highly reproducible and quantitative analyses could be performed of SAV and VAV. Although we endorse the findings of this study, there are some limitations to be addressed. First, the number of cases in this study is small. Yet, results of our study with respect to the correlations of fat distribution measurements are in line with previously published studies with more cases [19] (Additional file 1: Table S3). Second, as the CT scans were primarily performed to assess intra-abdominal tumor spread, SAV was not always completely visualized in the CT field of view (FOV) making exact quantification of SAV on some CT scans challenging. As a consequence, one patient who was believed to have more than 10% of SAV missing, was excluded from the SAV analyses. Furthermore, when restricting the analyses to patients in whom all SAV was included in the FOV, our findings remained the same. The estimated specific contribution of SAV to serum estradiol could thus have been even stronger if complete SAV had been included in the FOV of all patients. Third, our study and a previous study have found a strong correlation of SAV with VAV, which hampers identification of separate contributions of SAV and VAV with a general multiple linear regression model. Therefore, a commonality analysis was performed to separate the individual contributions of SAV and VAV as much as possible [19]. Fourth, analytical imprecision of estradiol at the low serum levels measured in this study, is relatively high. Therefore, it is difficult to identify statistically significant differences in small patient cohorts. Nevertheless, the estradiol levels in this study were comparable to results found in another study [17]. Since the blood samples donation protocol did not require a fasting or non fasting status, serum lipid levels might not be comparable, thereby hampering proper analysis of the relation with fat distribution [42]. Yet for estradiol, impact of fasting or time at blood withdrawal will have no effect on the results as postmenoapausal women have stable estradiol serum levels throughout the day [43]. A standardized protocol with blood sample donation on a fixed point of the day with uniformity in fasting status will enable more reliable analysis of the relation between lipids and fat distribution.

## Conclusions

This study shows that estradiol levels are correlated to BMI and fat distribution measurements in postmenopausal endometrial cancer patients. The subcutaneous fat contributes more to estradiol levels than the visceral fat indicating that subcutaneous fat might be relevant for endometrial cancer carcinogenesis. Validation of our findings in a study that evaluates all sex steroids implicated in the estrogen metabolism among patients with EC and premalignant lesions is warranted before definitive conclusions can be drawn. If high SAV is confirmed to play a particularly important role in the development of endometrial cancer, it may be relevant to explore it as an independent risk factor for developing cancer. Also the interplay between estradiol, hyperinsulinemia and inflammation in tumorgenesis should be clarified in order to determine the optimal strategy to prevent endometrial cancer.

## Additional file

Additional file 1: **Table S1.** Correlations of sex steroids. **Table S2.** Correlations between serum lipid levels. **Table S3.** Correlation between obesity markers including CT derived fat volumes. (XLSX 10 kb)

## Data Availability

The datasets used and during the current study are available from the corresponding author on reasonable request.

## Abbreviations

A4: Androstenedione
BMI: Body mass index
CT scan: Computed tomography scan
DHEAS: Dehydroepiandrosterone sulfate
E1: Estrone
E2: Estradiol
EC: Endometrial cancer
EEC: Endometrioid endometrial cancer
FIGO: International Federation of Gynaecology and Obstetrics
FOV: Field of view
HDL: High density lipoprotein
LDL: Low density lipoprotein
NEEC: Non endometrioid endometrial cancer
NHDL: Non-high density lipoprotein
SAV: Subcutaneous fat volume
T: Testosterone
TAV: Total abdominal fat volume
VAV: Visceral fat volume
VAV%: Percentage of visceral out of total abdominal fat volume ([VAV/TAV]x100)
WC: Waist circumference
